# *Chlorella vulgaris* Ameliorates Oxidative Stress and Improves the Muscle Regenerative Capacity of Young and Old Sprague-Dawley Rats

**DOI:** 10.3390/nu12123752

**Published:** 2020-12-07

**Authors:** Nurhazirah Zainul Azlan, Yasmin Anum Mohd Yusof, Suzana Makpol

**Affiliations:** 1 Department of Biochemistry, Faculty of Medicine, Level 17, Preclinical Building, Universiti Kebangsaan Malaysia Medical Centre, Jalan Yaacob Latif, Bandar Tun Razak, Cheras Kuala Lumpur 56000, Malaysia; nurhazirah@iium.edu.my; 2 Department of Basic Medical Sciences for Nursing, Kulliyyah of Nursing, Bandar Indera Mahkota Campus, International Islamic University Malaysia, Kuantan Pahang 25200, Malaysia; 3 Faculty of Medicine and Defense Health, Universiti Pertahanan Nasional Malaysia, Kem Sungai Besi, Kuala Lumpur 57000, Malaysia; yasmin.anum@upnm.edu.my

**Keywords:** sarcopenia, oxidative stress, muscle strength, muscle function, muscle mass

## Abstract

Muscle atrophy in ageing is a multifactorial degenerative process impacted by cellular ageing biology, which includes oxidative stress. *Chlorella vulgaris* is a coccoid green eukaryotic microalga rich in antioxidants. The aim of this study was to determine the effect of *C. vulgaris* in ameliorating oxidative stress, thus elucidating its mechanism in improving muscle mass, strength and function in young and old rats. Fifty-six male Sprague-Dawley (SD) rats aged 3 months (young) and 21 months (old) were divided into three groups: Group 1 (control) was given distilled water; Group 2 was treated with 150 mg/kg body weight (BW) of *C. vulgaris;* and Group 3 was treated with 300 mg/kg BW of *C. vulgaris* for three months. Grip and muscle strength and muscle integrity were determined on days 0, 30, 60, and 90 of treatment. Urine and blood were collected on days 0 and 90 of treatment for oxidative stress marker determination, while the gastrocnemius muscles were collected for muscle oxidative stress analysis. Increased grip strength of the front and hind paws was observed in young *C. vulgaris*-treated rats on days 30, 60, and 90 compared to the untreated control on the same days (*p* < 0.05). There was a significant increase in lean bone mineral content (BMC) in young rats treated with 300 mg/kg BW *C. vulgaris* compared to untreated rats on days 30 and 60. The fat mass was significantly decreased in young and old *C. vulgaris*-treated rats on day 90 compared to the untreated control. The total path was significantly increased for old rats treated with 300 mg/kg BW *C. vulgaris* on days 60 and 90 compared to day 0. Young and old *C. vulgaris*-treated rats demonstrated a significant decrease in urinary isoprostane F_2t_ and plasma creatine kinase-MM (CKMM) compared to the control on day 90. A significant decrease in malondialdehyde (MDA) and 4-hydroxyalkenal (HAE) levels were observed in young and old rats treated with *C. vulgaris*. *C. vulgaris* improved the muscle mass, strength, and function in young and old rats. This effect could be due to its potency in ameliorating oxidative stress in the skeletal muscle of young and old rats.

## 1. Introduction

Approximately 40% of the human body weight consists of skeletal muscle, which represents 50% to 75% of body proteins [1]. It accounts for 30% to 50% of the whole-body turnover of protein; hence, skeletal muscle is regarded as the body’s largest tissue [1]. Skeletal muscle performance diminishes with the progression of ageing in a state known as sarcopenia. Sarcopenia can be described as an age-linked decline in the strength, mass, and function of muscle, which is highly correlated with ageing [2]. The diverse factors of the degenerative process that influence muscle atrophy can be categorized into primary ageing, which is cellular ageing, and secondary ageing, which is affected by environmental, behavioral, and disease factors [3]. The most obvious and consistent changes related to the progression of ageing are a reduction in lean body mass and an increase in fat mass [4].

Sarcopenia can also be diagnosed based on the occurrence of both a slow gaiting speed and a low muscle mass [5]. The European Working Group on Sarcopenia in Older People (EWGSOP) suggested specifications for a sarcopenia diagnosis. This includes the detection of both a lower muscle mass, which can be quantified by dual-energy X-ray absorptiometry (DEXA), and the detection of the strength of the lower muscle and/or impaired performance, such as a slower speed of walking [6]. The muscle mass has been reported to decrease annually by 1% to 2% after 50 years of age, and muscle strength decreases by 1.5% per year between the ages of 50 and 60 years and 3% thereafter [7]. A previous longitudinal study reported that in elderly individuals aged 75 years old, the loss of muscle strength was 3.0% to 4.0% and 2.5% to 3.0% per year for men and women, respectively, and the loss of muscle mass was 0.80% to 0.98% and 0.64% to 0.70% per year for men and women, respectively [8].

The older population aged 70 to 80 years old is projected to increase due to an increased life expectancy [9]. Sarcopenia will lead to poor oxygen uptake, poor regulation of glucose, weakness and disability, loss of independence, decreased mobility, and increased risks of falls and fractures, and eventually contribute to morbidity and mortality [10,11,12]. Exercise and physical activity are effective approaches to alleviate sarcopenia that result in remarkable gains in muscle strength and power in elderly individuals [3]. Nutritional supplementation is another approach for sarcopenia management. Supplementation with essential amino acids and arginine has been reported to increase the body mass, function, and the strength of the muscle [13].

In this study, *Chlorella vulgaris* was investigated for its effects in the prevention of sarcopenia. In Japan, *C. vulgaris* powder is consumed as a supplement to replace a low intake of vegetables and fruits, as its composition is similar to that of green-yellow vegetables [14,15]. *C. vulgaris* has been reported to contain richly nutritive compounds such as carbohydrates, proteins, nucleic acids, minerals, β-carotene, chlorophyll-a and -b, lutein, tocopherol, ascorbic acid, retinol, and riboflavin [16,17]. Previous studies reported the antidiabetic [18,19] and anticancer effects of *C. vulgaris* [20,21,22]. *C. vulgaris* also exhibits higher antioxidant activities than the tocotrienol-rich fraction (TRF) and *Piper betle* [23]. Another study reported the antioxidant properties of *C. vulgaris* against induced liver cancer in rats were mediated by decreasing the oxidative stress markers SOD, MDA, and catalase [20].

*C. vulgaris* has shown potential as an anti-ageing compound by decreasing ageing biomarkers in senescent human diploid fibroblast (HDF) cells [24]. In our previous study on human skeletal muscle myoblast (HSMM) cells, it was observed that *C. vulgaris* was able to promote myoblast differentiation by increasing the fusion and maturation index. Additionally, *C. vulgaris* also decreased the expression of senescence biomarkers and increased the amount of positively stained myogenin, a protein that regulates the differentiation of muscle myoblasts [25]. *C. vulgaris* also demonstrated ameliorating outcomes on the myogenic regulatory factor (MRF) and muscle-specific miRNA (myomiR) expression of HSMM cells [26].

The antioxidant properties of *C. vulgaris* may promote muscle regeneration and further applications for sarcopenia prevention. Therefore, this research aimed to determine the effects of *C. vulgaris* in reviving muscle mass, strength, and function by focusing on its effect in ameliorating oxidative stress in the skeletal muscle of young and old Sprague-Dawley (SD) rats. Sprague-Dawley rats were chosen for their long lifespan, and that their age can be correlated with human ages. In this study, 3- and 21-month-old rats were given treatments for up to 3 months and were sacrificed at the ages of 6 and 24 months old, which correlates to human ages of 18 and 60 years old [27,28,29]. Therefore, the SD rats were able to manifest the characteristics of ageing in humans.

## 2. Materials and Methods

### 2.1. Animals

This research was performed in accordance with the "Guide for the Care and Use of Laboratory Animals" as postulated by the National Institute of Health (NIH), along with approval of the experimental design by the Universiti Kebangsaan Malaysia Medical Research and Innovation Secretariat (Project Code: FF-2016-318) and the Universiti Kebangsaan Malaysia Animal Ethics Committee (UKMAEC Approval Number: BIOC/PP/2016/SUZANA/27-JULY/770-SEPT.-2016-AUG.-2018). The SD rats were divided into two groups: 28 young male SD rats taken at 3 months old and 28 male aged SD rats taken at 21 months old. The rats used in this study weighed 250 to 400 g and were purchased from Nano Life Quest Sdn. Bhd. (Malaysia). Each age group of rats was further allotted into three groups: Group 1 was the control group given 1 mL of distilled water (*n* = 8), Group 2 was given 150 mg/kg body weight (BW) *C. vulgaris* (*n* = 10), and Group 3 was given 300 mg/kg BW *C. vulgaris* (*n* = 10) for three months. Each rat was randomly kept in individual Sealsafe^®^ Plus Rat IVC Green Line (TECHNIPLAST, Varese, Italy) cages in an animal care facility and allowed to acclimatize for one week before administration of the treatment. The rats were maintained in animal care facilities with a temperature of 24 °C and a 12-h light and dark cycle. The rats were also provided with rat pellets and water at all times (ad libitum), and wood shavings (Chipsi, Germany) were used as the bedding. The bedding was changed twice per week. The number of animals was chosen based on previous studies that used Wistar rats and diabetic type 2 Goto-Kakizaki [18,30].

### 2.2. *C. vulgaris* Preparation

The *C. vulgaris* Beijerinck strain 072 stock was acquired from the University of Malaya Algae Culture Collection (UMACC, Malaysia). Bold’s basal media (BBM) was used to grow the *C. vulgaris* stock with a 12-h light and dark cycle, and the stock was harvested by centrifugation at 1000 rpm. The centrifuged algae were weighed according to each rat’s body weight (BW) by diluting the algae in 1 mL distilled water prior to the treatments. The treatments were given as 1 mL of *C. vulgaris* extract by oral gavage once daily in the morning for 90 consecutive days. The rats were monitored daily for any sign of toxicity. Body weight was measured weekly, and the organs, including heart, liver, kidney, brain, and gastrocnemius muscle, were taken for the measurement of the relative organ weight after sacrifice.

### 2.3. Grip Strength Test

The front paw and hind paw grip strength were measured using a Bioseb Grip Strength Test (USA). The grip strength was used to determine the maximal peak force developed by a rat when it pulled out the metal bar. The machine was set up on a strong and stable table. The rat was allowed to grasp the metal bar by its front paws when it was pulled by the tail backwards in the horizontal plane. Peak tension, the force applied to the bar just before it lost its grip, was recorded in grams unit. The rats were pulled three times, and the machine recorded the highest peak tension. Then, the same procedure was repeated for the hind paw grip strength measurement.

### 2.4. Measurement of Bone Density

For the analysis of the computerized tomography of bone density, the entire body of the rat was scanned by applying dual energy X-ray absorptiometry (DXA) (Hologic Discovery W, Bedford, USA). The rats were anaesthetized 10 to 15 min before the procedure. The sedated rat was then placed on the X-ray platform, and scanning was performed within 3 min. As the scanning was performed, a large radiating scanning arm moved over the rats, and a low-dose X-ray beam was passed through the rats. The changes in radiation as it moved were converted by Hologic software for the quantification of bone mineral density (BMD), bone mineral content (BMC), lean BMC and fat mass for each rat.

### 2.5. Open Field Test

The rats were carried to the test room in its home cage and were handled by the base of their tails at all times. The open-field test was carried out in a square and black painted wooden box 60 cm × 60 cm. The rat was placed into one of the four corners facing towards the wall of the box and allowed to explore the field for 5 min, during which its movement was recorded by a video camera mounted to the ceiling. The rat was simultaneously assessed using HVS Image Software 2017 based on the parameters of interest, which include the total path length and the percentage of time moving. After the 5-min test, the rats were returned to their home cage, and the wooden box was wiped with 70% ethanol and permitted to dry between tests.

### 2.6. Urine Collection

Collection of urine was carried out from each rat on days 0 and 90 of treatment by spontaneous urination using clear plastic wrap. An empty clean cage was layered with cling wrap, and a single rat was allowed to roam inside the cage until urination. The rat was removed as soon as it urinated, and the voided urine was later aspirated with a pipette tip while avoiding contamination with any feces. A total of 1 mL of urine was collected and transferred to a microcentrifuge tube prior to being kept in a −80 °C freezer until further analysis.

### 2.7. Blood Collection

Blood was withdrawn from each rat on Day 0 and 90 of treatment by the orbital sinus collection methodology. The rat was anesthetized prior to blood collection and was handled with the thumb and forefinger of the left hand. The skin around the rat’s eye was pulled out until the eye bulged out, and a capillary tube was inserted with slight thumb pressure into the medial canthus of the eye at a 30 degree angle to the nose. Blood flowed through the capillary tube after puncturing through the tissue and plexus/sinus. A total of 3 mL of blood was collected into an EDTA tube before gently removing the capillary tube. The bleeding was then stopped by applying gentle pressure by the finger and wiping with sterile cotton. The collected blood was later centrifuged, and the plasma was kept in a −80 °C freezer until further analysis.

### 2.8. Euthanization of Animals

The anesthetic agent used in this study was KTX, which is a combination of ketamine, xylazine, and zoletil-50 (tiletamine and zolazepam). The KTX was administered based on 0.1 mL/250 g of rat body weight for each rat by intraperitoneal injection into the lower right quadrant of the abdomen due to its rapid onset, efficacy and minimal pain, fear and distress [31]. The rats were then left for approximately 30 min for the KTX to induce its sedative effect. This was observed by the clinical signs, including disorientation and a loss of consciousness, depression of respiratory or rapid irregular breathing, a progressive decline in heart rate and blood pressure, urination, and defecation. Once fully unconscious, the rats were euthanized by the decapitation method using a decapitator (Modiezham Sdn. Bhd., Kuala Lumpur, Malaysia).

### 2.9. Collection of Organs

All rats were fasted overnight on day 90 before sacrifice for necropsy examination. Organs such as the heart, liver, kidneys, brain, and gastrocnemius muscle were dissected from the sacrificed rats. All of the organs were washed with 90% normal saline to remove any adherent tissue and later weighed. The weight of the organs was taken as quickly as possible to avoid drying and was analyzed relative to the BW of the animals.

### 2.10. Analysis of Urinary Oxidative Stress

Urine samples collected from all rats were measured for 15-isoprostane F_2t_ activity by a urinary isoprostane ELISA kit (Oxford Biomedical Research, Oxford, USA) as specified by the manufacturer’s protocol. The sample was prepared by diluting 100 µL of urine with glucuronidase before being added to an anti-15-isoprostane F2t-coated well plate. The mixture was then mixed with 15-isoprostane F_2t_ horseradish peroxidase (HRP) conjugate followed by tetramethylbenzidine (TMB) substrate. The developed colour was quantified as the absorbance at 650 nm by a Multimode Plate Reader Enspire (Perkin Elmer, Singapore). The data obtained were calculated to acquire the concentration of isoprostanes (ng/mL) of each sample based on the standard curve plotted using the standard solution provided.

### 2.11. Analysis of Plasma Oxidative Stress

Plasma collected from each rat was measured for creatine kinase-MM (CKMM) activity by a Rat CK-MM ELISA Kit (Life Diagnostics Inc., West Chester, PA, USA) according to the manufacturer’s protocol. A total of 25 µL plasma was then diluted with diluent for sample preparation before being dispensed into anti-rat CK-MM antibody-coated microtiter plates. Enzyme conjugates were then added to each well followed by TMB reagent. The stop solution was added to stop the reaction and the plates were gently shaken before reading their absorbance at 450 nm with a Multimode Plate Reader Enspire (Perkin Elmer, Singapore). The results obtained were calculated to acquire the concentration of CKMM (ng/mL) of each sample based on the standard curve plotted using the standard solution provided.

### 2.12. Analysis of Skeletal Muscle Oxidative Stress

Lipid peroxidation was indicated by the measurement of malondialdehyde (MDA) and 4-hydroxyalkenals (HAE) by using a Bioxytech^®^ LPO-586^TM^ assay kit (OxisResearch, Oregon, USA) as specified by the manufacturer’s protocol. Briefly, 100 mg of gastrocnemius muscle was weighed and homogenized with PBS buffer containing 0.5 M BHT. The resulting clear supernatant homogenates were mixed with diluted R1 reagent followed by R2 reagent addition. The supernatant absorbance was quantified at 586 nm by a Multimode Plate Reader Enspire (Perkin Elmer, Singapore). The data obtained were calculated to acquire the concentration of MDA + HAE (μM) of each sample based on the standard curve plotted using the standard solution provided.

### 2.13. Statistical Analysis

All data are expressed as the mean ± SD, and a *p* value of <0.05 was considered statistically significant. SPSS software version 25 was used to carry out the statistical analysis. The data acquired were analysed by using one-way ANOVA followed by multiple comparisons by Tukey’s post hoc test.

## 3. Results

### 3.1. Body Weight and Relative Organ Weight

**Figure 1:**
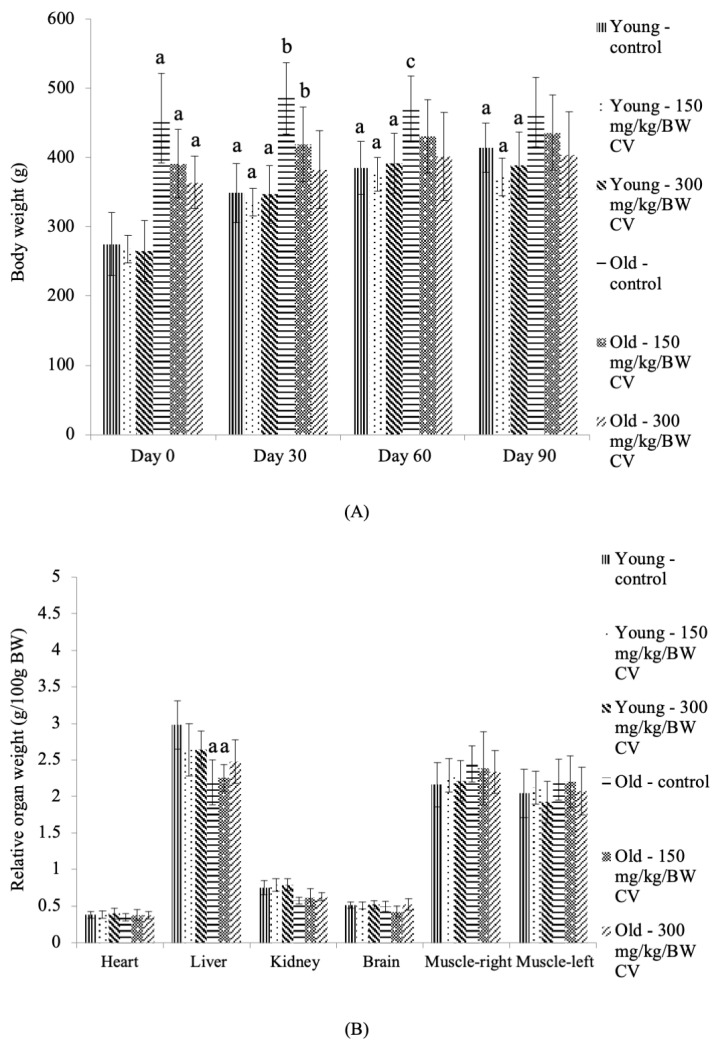
The (**A**) body weight and (**B**) relative organ weight of the young and old rats. The data are presented as the mean ± SD, *n* = 8 (control rats), *n* = 10 (*C. vulgaris*-treated rats). ^a^ *p* < 0.05 significantly different compared to the control young rats on Day 0, ^b^ *p* < 0.05 significantly different compared to the control young rats on Day 30, ^c^ *p* < 0.05 significantly different compared to the control young rats Day 60, with a post hoc Tukey HSD test. BW: Body weight; CV: *C. vulgaris*.

The body weights of the control young rats and young rats treated with *C. vulgaris* were significantly increased on days 30, 60, and 90 compared to the day 0 control young rats (*p* < 0.05) (Figure 1A). A corresponding increase was exhibited in the body weight of the control old rats and old rats treated with *C. vulgaris* compared to the control young rats on day 0 of treatment (*p* < 0.05). On day 30, the body weight of the control old rats and old rats treated with 150 mg/kg BW *C. vulgaris* was also significantly increased compared to that of the day 30 control young rats (*p* < 0.05). No significant changes were demonstrated in the body weight of the control and *C. vulgaris*-treated old rats on days 30, 60, and 90 compared to the day 0 control old rats.

Measurement of the organ weight showed that the liver and kidney weights of the control old rats and the old rats treated with *C. vulgaris* at 150 mg/kg BW were significantly decreased compared to the control young rats (*p* < 0.05) (Figure 1B).

### 3.2. Measurement of Grip Strength

**Figure 2:**
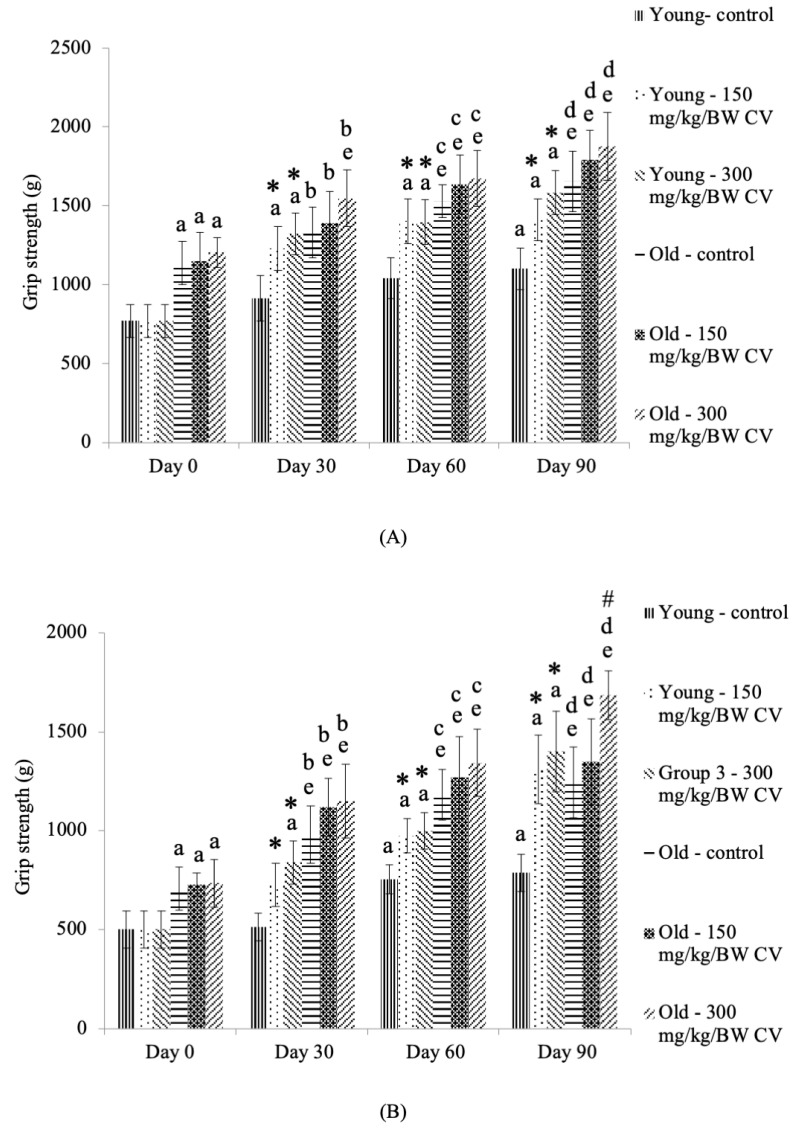
The grip strength of (**A**) the front paws and (**B**) the hind paws of young and old rats. The data are presented as the mean ± SD, *n* = 8 (control rats), *n* = 10 (*C. vulgaris*-treated rats). ^*^ *p* < 0.05 significantly different compared to the control young rats on the respective day, ^#^ *p* < 0.05 significantly different compared to the control old rats on the respective day, ^a^ *p* < 0.05 significantly different compared to the control young rats on Day 0, ^b^ *p* < 0.05 significantly different compared to the control young rats on Day 30, ^c^ *p* < 0.05 significantly different compared to the control young rats on Day 60, ^d^ *p* < 0.05 significantly different compared to the control young rats on Day 90, ^e^ *p* < 0.05 significantly different compared to the control old rats on Day 0, with a post hoc Tukey HSD test. BW: Body weight; CV: *C. vulgaris.*

The grip strength of the front paws of the control young rats was significantly increased on day 90 of treatment compared to the day 0 control young rats (*p* < 0.05) (Figure 2A). *C. vulgaris* treatment of the young rats showed a significant increase in front paw grip strength on days 30, 60, and 90 compared to day 0 (*p* < 0.05). The treatment of young rats with *C. vulgaris* significantly increased the grip strength of the front paws on days 30, 60, and 90 compared to that of the control young rats on the respective days of treatment (*p* < 0.05).

The front paw grip strength of the old rats was significantly increased on days 0, 30, 60, and 90 of treatment compared to that of the control young rats on the respective days of treatment (*p* < 0.05) (Figure 2A). A similar increase in front paw grip strength was observed in control old rats and old rats treated with *C. vulgaris* on days 60 and 90 of treatment compared to day 0 control old rats (*p* < 0.05). *C. vulgaris* treatment at 300 mg/kg BW significantly increased the grip strength of the front paws of old rats on day 30 of treatment compared to the day 0 control old rats (*p* < 0.05).

The grip strength of the hind paws of the control young rats and the young rats treated with *C. vulgaris* was significantly increased on Day 60 and Day 90 of treatment compared to the Day 0 control young rats (*p* < 0.05) (Figure 2B). On day 30 of treatment, the grip strength of the hind paws of the young rats treated with 300 mg/kg BW *C. vulgaris* was significantly increased compared to that of the young rats on day 0 (*p* < 0.05). Treatment with *C. vulgaris* significantly increased the grip strength of the hind paws of the young rats on days 30, 60, and 90 compared to that of the respective control (*p* < 0.05).

The hind paw grip strength of the old rats was significantly increased on days 0, 30, 60, and 90 of treatment compared to that of the control young rats on the respective days of treatment (*p* < 0.05) (Figure 2B). A similar increase in hind paw grip strength was observed in the control old rats and the old rats treated with *C. vulgaris* on days 30, 60, and 90 of treatment compared to the day 0 control old rats (*p* < 0.05). Treatment with 300 mg/kg BW *C. vulgaris* significantly increased the hind paw grip strength of the old rats on day 90 compared to its control (*p* < 0.05).

### 3.3. Measurement of Muscle and Bone Integrity

**Figure 3:**
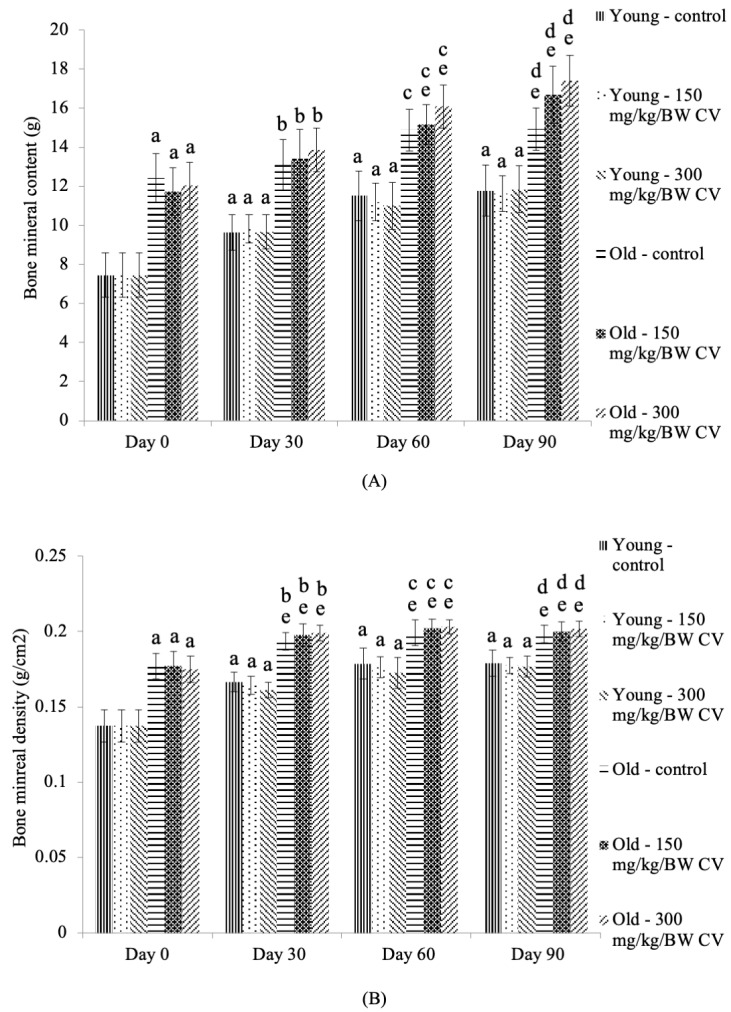

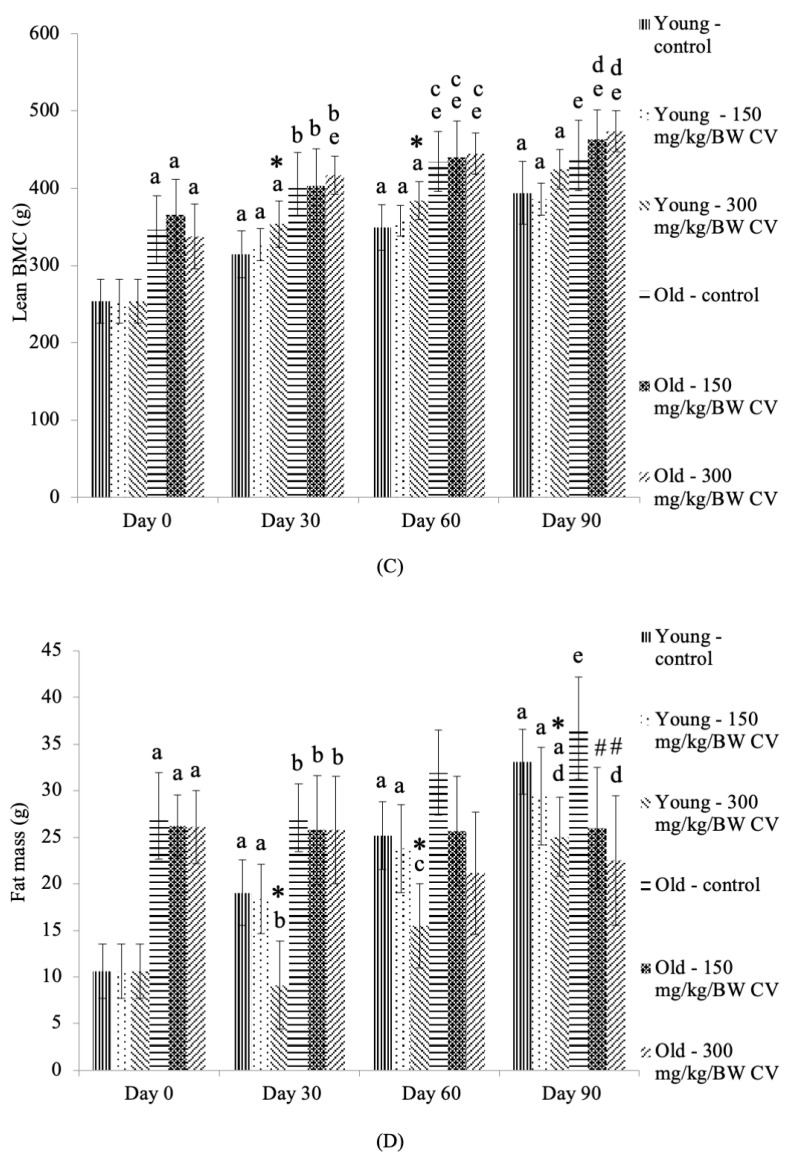
The (**A**) bone mineral content (BMC), (**B**) bone mineral density (BMD), (**C**) lean BMC and (**D**) fat mass of the young and old rats. Data are presented as the mean ± SD, *n* = 8 (control rats), *n* = 10 (*C. vulgaris*-treated rats). ^*^ *p* < 0.05 significantly different compared to the control young rats on the respective day, ^#^ *p* < 0.05 significantly different compared to the control old rats on the respective day, ^a^ *p* < 0.05 significantly different compared to the control young rats on Day 0, ^b^ *p* < 0.05 significantly different compared to the control young rats on Day 30, ^c^ *p* < 0.05 significantly different compared to the control young rats on Day 60, ^d^ *p* < 0.05 significantly different compared to the control young rats on Day 90, ^e^ *p* < 0.05 significantly different compared to the control old rats on Day 0, with a post hoc Tukey HSD test. BW: Body weight; CV: *C. vulgaris.*

The BMCs of the control young rats and the young rats treated with *C. vulgaris* were significantly increased on days 30, 60, and 90 compared to the day 0 control young rats (*p* < 0.05) (Figure 3A). The BMCs of the control old rats and the old rats treated with *C. vulgaris* were significantly increased on days 0, 30, 60, and 90 of treatment compared to the control young rats on the respective days of treatment (*p* < 0.05). A similar increase in BMCs was observed in old rats treated with *C. vulgaris* on days 60 and 90 compared to the day 0 control old rats (*p* < 0.05). The BMC was also increased in the control old rats on day 90 compared to the day 0 control old rats (*p* < 0.05).

The BMD of the control young rats and the young rats treated with *C. vulgaris* was significantly increased on days 30, 60, and 90 compared to the day 0 control young rats (*p* < 0.05) (Figure 3B). The BMD of the control old rats and old rats treated with *C. vulgaris* was significantly increased on days 0, 30, 60, and 90 of treatment compared to the control young rats on the respective days of treatment (*p* < 0.05). A similar increase in BMD was observed in the control old rats and the old rats treated with *C. vulgaris* on days 30, 60, and 90 compared to the day 0 control old rats (*p* < 0.05).

The lean BMC of the control young rats and young rats treated with *C. vulgaris* was significantly increased on days 30, 60, and 90 compared to the day 0 control young rats (*p* < 0.05) (Figure 3C). Treatment with 300 mg/kg BW *C. vulgaris* in the young rats significantly increased their lean BMC on days 30 and 60 of treatment compared to that of the respective control (*p* < 0.05). The lean BMCs of the control old rats and the old rats treated with *C. vulgaris* were significantly increased on days 0, 30, and 60 of treatment compared to the control young rats on the respective days of treatment (*p* < 0.05). A similar increase in lean BMCs was observed in the old rats treated with *C. vulgaris* on day 90 of treatment compared to the day 90 control young rats (*p* < 0.05). The lean BMC was significantly increased in the control old rats and the old rats treated with *C. vulgaris* on days 60 and 90 compared to the day 0 control old rats (*p* < 0.05). On day 30 of treatment, the lean BMC of the old rats treated with 300 mg/kg BW *C. vulgaris* was significantly increased compared to that of the day 0 control old rats (*p* < 0.05).

The lean fat mass of the control young rats and the young rats treated with 150 mg/kg BW *C. vulgaris* was significantly increased on days 30 and 60 compared to the day 0 control young rats (*p* < 0.05) (Figure 3D). A similar increase in the lean fat mass of the control young rats and the young rats treated with *C. vulgaris* on day 90 of treatment was seen as compared to the day 0 control young rats (*p* < 0.05). Treatment with 300 mg/kg BW *C. vulgaris* significantly decreased the lean fat mass in the young rats on days 30, 60, and 90 of treatment compared to their respective control (*p* < 0.05). The lean fat mass of the control old rats and the old rats treated with *C. vulgaris* was significantly increased on days 0 and 30 compared to the control young rats on the respective days of treatment (*p* < 0.05). However, treatment with 300 mg/kg BW *C. vulgaris* decreased the lean fat mass of the old rats on day 90 compared to the day 90 control young rats (*p* < 0.05). A similar decrease in fat mass was observed in old rats treated with *C. vulgaris* on day 90 compared to the day 90 control old rats (*p* < 0.05).

### 3.4. Measurement of Muscle Function

**Figure 4:**
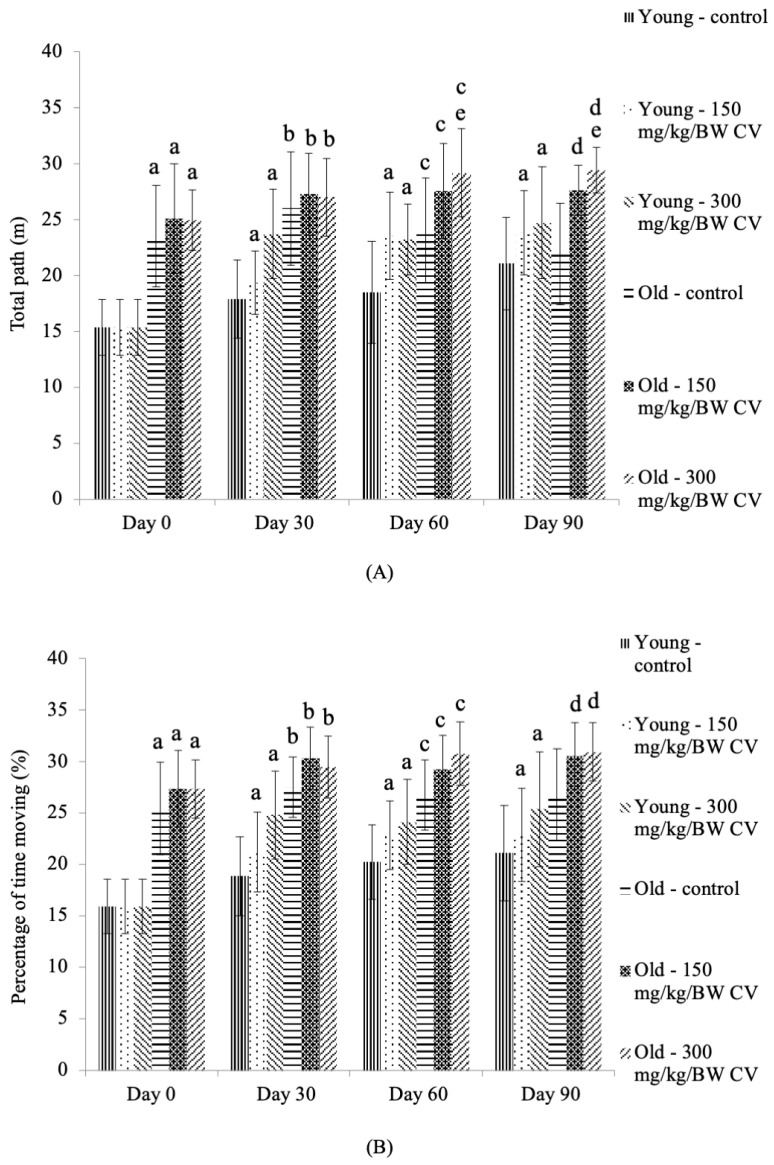
The (**A**) total path and (**B**) the percentage of time spent moving by the young and old rats. The data are presented as the mean ± SD, *n* = 8 (control rats), *n* = 10 (*C. vulgaris*-treated rats). ^a^ *p* < 0.05 significantly different compared to the control young rats on Day 0, ^b^ *p* < 0.05 significantly different compared to the control young rats on Day 30, ^c^ *p* < 0.05 significantly different compared to the control young rats on Day 60, ^d^ *p* < 0.05 significantly different compared to the control young rats on Day 90, ^e^ *p* < 0.05 significantly different compared to the control old rats on Day 0, with a post hoc Tukey HSD test. BW: Body weight; CV: *C. vulgaris.*

In the open field test, the total path of the young rats treated with *C. vulgaris* was significantly increased on days 30, 60, and 90 compared to the day 0 control young rats (*p* < 0.05) (Figure 4A). The total path of the control old rats and the old rats treated with *C. vulgaris* was significantly increased on days 0, 30, and 60 compared to the control young rats on the respective days of treatment (*p* < 0.05). A similar increase in the total path was observed among old rats treated with *C. vulgaris* on day 90 compared to the day 90 control young rats (*p* < 0.05). Treatment with 300 mg/kg BW *C. vulgaris* significantly increased the total path of the old rats on days 60 and 90 compared to the day 0 control old rats (*p* < 0.05).

The percentage of time spent moving by young rats treated with *C. vulgaris* was significantly increased on days 30, 60, and 90 compared to the day 0 control young rats (*p* < 0.05) (Figure 4B). The percentage of time spent moving by the control old rats and the old rats treated with *C. vulgaris* was significantly increased on days 0, 30, and 60 compared to the control young rats on the respective days of treatment (*p* < 0.05). A similar increase in the percentage of time spent moving was observed in the old rats treated with *C. vulgaris* on day 90 compared to the day 90 control young rats (*p* < 0.05).

### 3.5. Levels of Oxidizing Stress Markers in the Tissue, Plasma, and Urine

**Figure 5:**
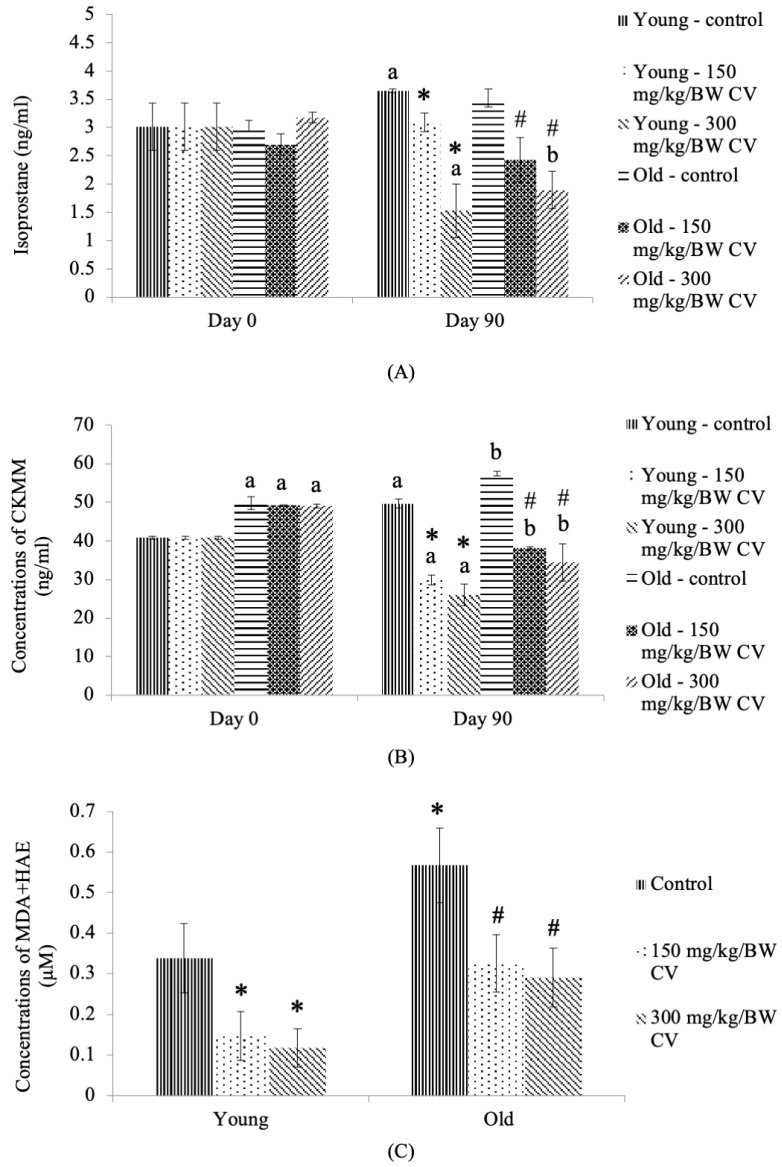
The (**A**) urinary oxidative stress, (**B**) plasma oxidative stress and (**C**) skeletal muscle oxidative stress of young and old rats. Data are presented as the mean ± SD, *n* = 8 (control rats), *n* = 10 (*C. vulgaris*-treated rats). ^*^p < 0.05 significantly different compared to the control young rats on Day 90, ^#^ *p* < 0.05 significantly different compared to the control old rats on Day 90, ^a^ *p* < 0.05 significantly different compared to the control young rats on Day 0, ^b^ *p* < 0.05 significantly different compared to the control old rats on Day 0, with a post hoc Tukey HSD test. BW: Body weight; CV: *C. vulgaris.*

The isoprostane F_2t_ urinary oxidative stress level of the control young rats was significantly increased on day 90 compared to the day 0 control young rats (*p* < 0.05) (Figure 5A). On day 90, *C. vulgaris* treatment significantly decreased the isoprostane F_2t_ urinary oxidative stress level of the young rats compared to the day 90 control young rats (*p* < 0.05). A similar decrease was observed in the isoprostane F_2t_ urinary oxidative stress level of young rats treated with 300 mg/kg BW *C. vulgaris* on day 90 of treatment compared to the day 0 control young rats (*p* < 0.05).

On day 90, *C. vulgaris* treatment decreased the isoprostane F_2t_ urinary oxidative stress level of old rats compared to that of the day 90 control old rats (*p* < 0.05) (Figure 5A). A similar decrease was observed in the isoprostane F_2t_ urinary oxidative stress level of old rats treated with 300 mg/kg BW *C. vulgaris* on day 90 of treatment compared to the day 0 control old rats (*p* < 0.05).

The level of plasma CKMM was significantly increased in the control young rats on day 90 of treatment compared to the day 0 control young rats (*p* < 0.05) (Figure 5B). Treatment with *C. vulgaris* significantly decreased the level of plasma CKMM in young rats on day 90 compared to the day 0 and day 90 control young rats (*p* < 0.05). The level of plasma CKMM was significantly increased in the control old rats and the old rats treated with *C. vulgaris* on day 0 of treatment compared to the day 0 control young rats (*p* < 0.05). A similar increase in the level of plasma CKMM was observed in the control old rats on day 90 of treatment compared to the day 0 control old rats (*p* < 0.05). Treatment with *C. vulgaris* significantly decreased the level of plasma CKMM in old rats on day 90 compared to the day 0 and day 90 control old rats (*p* < 0.05).

A significant increase in the MDA and HAE levels in the control old rats compared to the control young rats (*p* < 0.05) was observed (Figure 5C). Treatment with *C. vulgaris* significantly decreased the levels of tissue MDA and HAE in both the young and old rats compared to the control young and control old rats, respectively (*p* < 0.05).

## 4. Discussion

Ongoing research on sarcopenia mainly focuses on alleviating the disease and reducing the economic burden resulting from this overwhelming condition in the elderly population. This disease is characterized by an increased risk of falls and fracture due to a decrease in muscle strength and mass, a decrease in BMD and limited mobility [32]. Improvements in the characteristics of sarcopenia may produce favourable effects towards modulating sarcopenia in the elderly. In this study, rats in two different age groups representing young and elderly humans were used to measure muscle function. The results of this study showed that the body weight of the young rats was significantly increased with an increasing number of days, suggesting the effect of age on the body weight of the young rats. However, no significant difference was seen in the body weight of the old rats with an increasing number of days. This could be due to the slower rate of muscle regeneration in old rats or the growth process in old rats reaching a plateau. In a previous study, the body weight of the C57BL/6 mice was reported to increase significantly until 12 months of age (mid age), which then reached a plateau, with no increase in body weight observed [33]. In sarcopenia, overall body weight may not be affected, and it may not lead to a net body weight loss due to increased deposition of lipids within the muscle fibers and fat tissue, particularly intra-abdominal fat. As a result, the body weight can be variable due to variations in muscular mass, adipose tissue and bone mass [34,35].

No significant difference was observed in the relative organ weight of the heart, brain, and muscle for all young groups treated with *C. vulgaris*, suggesting that no toxicity of *C. vulgaris* occurred. A previous study also reported no toxicity or mortality in female SD rats treated with *C. vulgaris* by using the Organization for Economic Cooperation and Development (OECD) Guideline 420 [36]. However, a significant decrease in the liver and kidney relative organ weight was observed in the control old rats and the old rats treated with *C. vulgaris* compared to the control young rats. This finding was similar to a previous finding in C57BL/6 mice, which reported that the kidney weight reached a plateau at 23 to 28 months (old mice), and a significant decrease in the liver weight was observed at 23 to 28 months of age and 30 months of age (very old) [33].

The grip strength test has been used for the assessment of the effects of drugs, toxins, and chemical substances on muscle function and it is also suitable to measure the muscle strength in clinical settings [37,38,39]. This test is usually performed to quantify muscular strength in age-associated neurodegenerative diseases such as stroke and Parkinson’s disease [40,41,42] to measure any alterations in motor coordination [40]. The results of this study showed that the front paw grip strength of young rats was significantly increased with an increasing number of days, indicating the effect of age in promoting grip strength in young rats. Treatment with *C. vulgaris* was found to increase the grip strength of young rats, indicating the ability of *C. vulgaris* to improve the muscle function in young rats. Changes in grip strength values, such as low scores, indicate a low muscular strength, and they therefore act as evidence of motor neurotoxicity, while an increased grip strength indicates an improvement of muscle function [43]. A similar observation was found in old rats, whereby the front paw grip strength of the old rats was significantly increased with an increasing number of days, suggesting an effect of age in promoting muscle function. However, *C. vulgaris* treatment did not result in any significant impact on the grip strength of the front paws of the old rats.

The hind paw grip strength of the young and old rats gave a similar pattern of increment as the grip strength of the front paws. The hind paw grip strength of the young and old rats was significantly increased with an increasing number of days. Treatment with *C. vulgaris* at one month and two months was found to increase the hind paw grip strength of young rats compared to the untreated control. In addition, *C. vulgaris* treatment for three months improved the grip strength of the hind paws of both the young and old rats, confirming the beneficial effect of *C. vulgaris* in improving muscle function.

A DXA scan is an effortless, reproducible, and precise method to analyze the composition of bodies. A previous study reported that the BMD and total body soft-tissue composition and major subregions measured with DXA demonstrated a precise composition analysis even at different times of measurement with low radiation exposure, and it has been used for the assessment of muscle mass in elderly men [44,45,46]. The results from this study demonstrated that the BMD, BMC, and lean BMC were significantly increased with an increasing number of days in both young and old rats. The fat mass, however, was significantly increased with an increase in the number of days only in young rats, and no difference was observed in the old rats. Another previous study also reported the use of DXA in assessing the skeletal muscle mass, strength and quality in older populations and showed that muscle mass loss is related to a decrease in muscle strength in older adults. However, the muscle strength decline is much faster than the muscle mass decline [46]. Treatment with *C. vulgaris* at 300 mg/kg BW for one month and two months in this study was found to increase the lean BMCs in young rats. No similar effect was observed in old rats. Treatment with 300 mg/kg BW *C. vulgaris* also decreased the fat mass in young rats. In old rats, *C. vulgaris* decreased the fat mass, indicating that *C. vulgaris* has the capability of improving muscle strength by modulating the BMC and fat mass in the body. This also indicates that *C. vulgaris* has the potential to increase the skeletal muscle mass, as greater skeletal muscle mass is related to greater BMC [47].

Increased muscle mass and decreased fat mass may aid in reversing the effects of sarcopenia and ageing. A similar result was observed in a previous study of 19-month-old Wistar Han rats that were treated with espindolol, a small-molecule anabolic catabolic transforming agent (ACTA). Espindolol was found to increase muscle mass and decrease fat mass by reducing p38 phosphorylation [34]. Increased p38 phosphorylation was reported to occur in the branchial muscle of old rats, resulting in decreased muscle-specific gene expression. Cellular stress is one of the factors that can cause increased p38, which leads to its activation and affects muscle mass [48]. Therefore, *C. vulgaris* may improve muscle mass and decrease fat mass through the inactivation of p38 via its antioxidant properties. A previous study reported that esomeprazole, a treatment for stress ulcers, inactivates the p38 MAPK pathway by its antioxidative effect [49].

The open field test monitoring system is a functional instrument for examining the impairment in locomotion in animal models with neuromuscular disease and the potency of therapeutic drugs, which could help improve muscle function and locomotion. Animals with a decreased total path distance and movement time are associated with decreased muscle function because these animals will be less active and exhibit reduced ambulatory activity [50]. The total path for young 300 mg/kg BW *C. vulgaris*-treated rats was significantly increased compared to that of untreated rats on day 30. For old rats, 300 mg/kg BW *C. vulgaris* significantly increased the total path on days 60 and 90 compared to untreated old rats on day 0. However, no significant differences were demonstrated in the percentage of time spent moving in old untreated and treated rats, which might be due to less movement by the old rats. A previous study reported a significant decrease in total distance travelled, horizontal and vertical activity, and time of movement and rest in 30- to 33-week-old dystrophia muscularis 2 Jackson (B6. WK-Lama2^dy−2J^/J) mice, a mouse model of laminin mutations that exhibit paralysis of the hindlimbs associated with dystrophic changes and demyelination of nerves in the skeletal muscle [51].

Excessive reactive oxygen species (ROS) production occurs when the production rate of free radicals overcomes the capacity of antioxidant defense in the body. Excess ROS in the body can cause oxidative stress, resulting in damage to mitochondrial DNA and the loss of organelle functions, and this may lead to accelerated ageing. This damage accumulates with increasing age in almost all organs, including skeletal muscle. The increase in ROS and reactive nitrogen species (RNS) production has been reported to have an impact on signalling pathways that are in charge of the synthesis of protein and proteolysis in skeletal muscle; therefore, oxidative stress can be one of the mechanisms involved in the development of sarcopenia [52,53,54].

The measurement of urinary F_2t_ isoprostanes has been described in a previous study as one of the reliable indicators to ascertain oxidative stress in vivo [55,56]. The F_2t_ isoprostanes are prostaglandin PGF_2α_ isomers that are produced in vivo from the free radical-initiated peroxidation of arachidonic acid, independent of cyclooxygenase enzymes [55,57,58,59]. Another oxidative stress marker in ageing that has been widely used is creatine kinase (CK). Creatine kinase (CK) is also recognized as creatine phosphokinase and it occurs in three isoenzymes, CK1, CK2, and CK3. The isoenzyme CK3 contains MM subunits and is found in skeletal muscle, and CK-MM is a practical biomarker for skeletal muscle injury [60]. In 1997, Roserberg was the first researcher to define sarcopenia. He reported that creatinine, a breakdown product of creatine phosphate in the muscle, can be used to measure the muscle mass. Creatinine was found to decrease with increasing age, indicating creatine kinase-MM as a useful marker in skeletal muscle injury resulting from the presence of excessive oxidative stress [61]. The CKMM released to the bloodstream indicates the manifestation of both mechanical and metabolic disturbances within the sarcomere, which also reflects the integrity, stability and function of the plasma membrane [62].

A previous study reported that there was an age-associated increase in lipid peroxide concentration, with the MDA level being significantly higher in the 50- to 59-year-old age group than in the 30- to 39-year-old age group in a human study [63]. Another study reported a significant increase in MDA levels along with muscle injury biomarkers, such as CK and lactate dehydrogenase (LDH), in older subjects (65.1 ± 3.5 years old) compared to young subjects (20.3 ± 2.8 years old) [64]. Sarcopenia also occurs when the relative skeletal muscle mass index (RASM) for women was <5.67 kg/m^2^ and for men it was <7.25 kg/m^2^, and the plasma ratio of MDA/HNE was significantly increased in sarcopenic patients compared to nonsarcopenic patients [65]. It is well known that ageing is accompanied by increased lipid peroxidation due to a decreased antioxidant defense resulting from increased ROS production, indicating skeletal muscle oxidative injury [64]. In this study, CKMM and MDA + HAE were significantly higher in untreated old rats than in untreated young rats, and *C. vulgaris* treatment in this study was observed to decrease the urinary isoprostane F2t and MDA + HAE levels in both the young and old rats compared to the respective untreated controls on day 90. This observation may be attributed to the antioxidant properties of *C. vulgaris.*

This finding was in association with a previous study that used a *Chlorella*-supplemented diet in the treatment of muscle-specific mitochondrial aldehyde dehydrogenase 2 activity-deficient mice (ALDH2*2 Tg mice) characterized by age-associated muscle atrophy, as in sarcopenia. This study reported that urinary isoprostane levels declined in ALDH2*2 Tg mice fed a diet supplemented with *Chlorella* for four months and that MDA + HAE levels declined in LDH2*2 Tg mice fed a diet supplemented with *Chlorella* for six months [66]. The findings of this study also demonstrated that the plasma oxidative stress of creatine kinase-MM was significantly decreased in both young and old rats treated with *C. vulgaris* compared to its respective control on day 90. This observation may signify that the consumption of *C. vulgaris* helps reduce oxidative stress, as ascertained by oxidative stress markers.

A previous study showed that HDFs induced with oxidative stress by hydrogen peroxide demonstrated DNA damage and a decreased telomere length and telomerase activity. However, *C. vulgaris* treatment modulated the effects of hydrogen peroxide in HDF cells obtained from both young and old donors [67]. Another study reported that the consumption of a *Chlorella*-supplemented diet by ALDH2*2 Tg mice improved their skeletal muscle atrophy by increasing the size of their muscle cells. Additionally, the relative cross-sectional areas of the gastrocnemius muscle cells were negatively correlated with the expression levels of MDA and HAE in ALDH2*2 Tg mice. This study also suggested that a *Chlorella*-supplemented diet prevented mitochondrial dysfunction in the gastrocnemius muscle, as demonstrated by the decline in cytochrome *c* oxidase activity [66].

**Figure 6:**
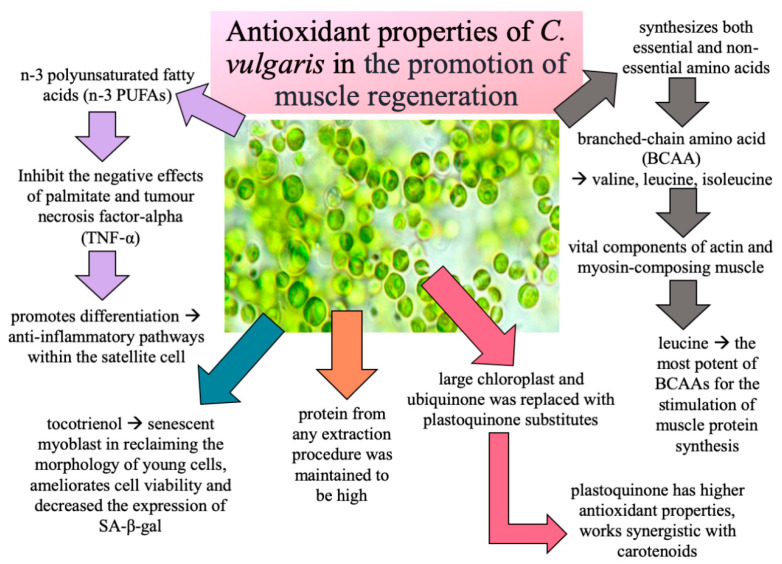
The antioxidant properties of *C. vulgaris* and its effect in promoting muscle regeneration [17,66,67,68,69,70,71,72,73,74,75,76].

Other previous studies have reported the promotion of the differentiation of human myoblast cells in young and senescent myoblasts, which consequently helps in the reversal of ageing and may be beneficial for the treatment of age-related muscular disease [25,26]. In this study, *C. vulgaris* showed its potential in promoting muscle function, as indicated by an increased muscle mass and strength in young and old rats. Decreases in urinary isoprostane F_2t_, plasma CKMM and the skeletal muscle oxidative stress markers MDA and HAE were also demonstrated in young and old rats treated with *C. vulgaris*. The antioxidant features of *C. vulgaris* may be held accountable in promoting differentiation in both muscle myoblast cells and animal studies. Figure 6 summarizes the antioxidant components of *C. vulgaris* that have been reported in previous studies and their effects in promoting muscle regeneration.

## 5. Conclusions

*C. vulgaris* supplementation is beneficial as an anti-ageing agent in young and old rats due to its effect in promoting muscle function, thus enhancing muscle performance. These effects could be due to its antioxidant properties and its ability to ameliorate oxidative stress. This finding may indicate its potential in reducing the severity of sarcopenia and may also be beneficial for other age-related muscular atrophy conditions and other muscular diseases.
